# Community-acquired Pneumonia among Elderly Patients Admitted to the Department of Medicine in a Tertiary Care Centre: A Descriptive Cross-sectional Study

**DOI:** 10.31729/jnma.8202

**Published:** 2023-06-30

**Authors:** Lochan Karki, Milan Khadka, Santosh Joti, Siddhant Adhikari, Rama Tamrakar, Milan Purna Oli, Poonam K C, Shrinkhala Maharjan, Shristi Maharjan, Rabin Basnet, Shanta Chauhan, Prapti Basnet

**Affiliations:** 1 Department of Medicine, National Academy of Medical Sciences, Mahaboudha, Kathmandu, Nepal; 2 Silverline Hospital, Balaju, Kathmandu, Nepal; 3 Lubhoo Primary Health Care Centre, Mahalaxmi, Lalitpur, Nepal; 4 Godawari Midcity Hospital, Satdobato, Lalitpur, Nepal; 5 Ganeshman Singh Memorial Hospital and Research Center, Ring Road, Lalitpur, Nepal; 6 Annapurna Neurological Institute and Allied Sciences, Maitighar, Kathmandu, Nepal; 7 Karuna Hospital, Budhanilkantha, Kathmandu, Nepal; 8 Department of Tele Medicine Bir Hospital, Mahaboudha, Kathmandu, Nepal; 9 Department of Emergency Medicine, Himal Hospital Gyaneshwor, Kathmandu, Nepal; 10 Bir Hospital, Mahaboudha, Kathmandu, Nepal

**Keywords:** *elderly*, *pneumonia*, *prevalence*, *tertiary care center*

## Abstract

**Introduction:**

Community-acquired pneumonia is an acute infection of lung tissue in an immunocompetent patient who have not recently been hospitalized or has been hospitalized for less than 48 hours and acquired it in the community. It continues to have a substantial effect on the elderly, who are impacted more often and severely than younger groups. It is the third most common hospital diagnosis in adults over the age of 65 years, and the sixth major cause of mortality in developed nations. The aim of this study was to find out the prevalence of community-acquired pneumonia among elderly patients admitted to the Department of Medicine in a tertiary care centre.

**Methods:**

A descriptive cross-sectional study was done in the Department of Medicine in a tertiary care centre after obtaining ethical approval from the Institutional Review Committee (Reference number: 465/2079/80). Data from 11 December 2021 and 1 December 2022 were collected between 1 April 2023 to 15 April 2023 from the hospital records. Data on community-acquired pneumonia in the elderly was collected from the hospital records. Convenience sampling method was used. Point estimate and 95% Confidence Interval were calculated.

**Results:**

Among 385 patients, community-acquired pneumonia was seen in 14 (3.64%) (1.77-5.51, 95% Confidence Interval) with a mean age of 70.57±10.21 years.

**Conclusions:**

The prevalence of community-acquired pneumonia among elderly patients was found to be lower compared to other studies conducted in similar settings.

## INTRODUCTION

Pneumonia is a lung parenchymal infection and is divided into four types-community-acquired pneumonia (CAP), hospital-acquired pneumonia, healthcare-associated pneumonia, and ventilator-associated pneumonia.^1-3^

Community-acquired pneumonia is an acute infection of lung tissue in an immunocompetent patient who has not recently been hospitalized or has been hospitalized for less than 48 hours and acquired it in the community.^3,4^ It has a more substantial effect on the elderly than on younger groups.^5^ It is the third most common hospital diagnosis in adults over the age of 65 years, and the sixth major cause of mortality in developed nations.^6^ Extrapulmonary manifestations (delirium, worsening of a chronic confusion or fall) are more common than pulmonary findings (fever, expectoration, difficulty breathing) in elderly patients with pneumonia making diagnosis difficult and delaying prompt recognition and treatment of CAP.^7^

The aim of this study was to find out the prevalence of community-acquired pneumonia among elderly patients admitted to the Department of Medicine in a tertiary care centre.

## METHODS

This descriptive cross-sectional study was conducted among the elderly in the Department of Medicine, National Academy of Medical Sciences, Mahaboudha, Kathmandu, Nepal after obtaining ethical approval from the Institutional Review Committee (IRC) (Reference number: 465/2079/80). Data from 11 December 2021 and 1 December 2022 were collected between 1 April 2023 to 15 April 2023 from the hospital records. All the elderly patients more than 65 years admitted during the study duration were included. Incomplete data, patients discharged on leaving against medical advice (LAMA) and clear alternative diagnosis were excluded. Convenience sampling method was used. The sample size was calculated using the following formula:


$$\begin{array}{ll} \text{n} & ={\text{Z}}^{2}\times \frac{\text{p}\times \text{q}}{{\text{e}}^{\text{2}}}\\ & =1.96^{2}\times \frac{0.50\times 0.50}{0.05^{2}}\\ & =385 \end{array}$$

Where,

n = minimum required sample size
Z = 1.96 at 95% Confidence Interval (CI)
p = prevalence taken as 50% for maximum sample size calculation
q = 1-p
e = margin of error, 5%

The minimum required sample size was 385 and we took 385 samples.

Community-acquired pneumonia is an acute infection of lung tissue in an immunocompetent patient who has not recently been hospitalized or has been hospitalized for less than 48 hours and acquired it in the community.^3,4^ Ageing is generally assessed by chronological age, and a person aged 65 or older is often referred to as elderly.^8^

Data was entered in Microsoft Excel 2016 and was analyzed in IBM Statistics SPSS 26.0. Point estimate and 95% CI were calculated.

## RESULTS

Among 385 patients, community acquired pneumonia was seen in 14 (3.64%) (1.77-5.51, 95% Confidence Interval) with the mean age of 70.57±10.21 years. The mean hospital stay was found to be 7.42±3.41 days ranging from 2 days to 13 days. Among 14 patients, 11 (78.57%) recovered and were discharged whereas 3 (21.42%) died during the hospital stay (Table 1).

**Table 1:** Demographic characteristics of the elderly community-acquired pneumonia patients (n= 14).

Parameters		n (%)
Gender	Male	6 (42.86)
	Female	8 (57.14)
Age group (years)	65-79	10 (71.43)
	80-94	3 (21.43)
	≥ 95	1 (7.14)
Ethnicity	Newar	5 (35.71)
	Chhetri	2 (14.29)
	Tamang	2 (14.29)
	Brahmin	1 (7.14)
	Others	4 (28.57)

Nine (64.29%) patients had a history of smoking and 4 (28.57%) had a history of alcohol intake.

**Figure 1:**
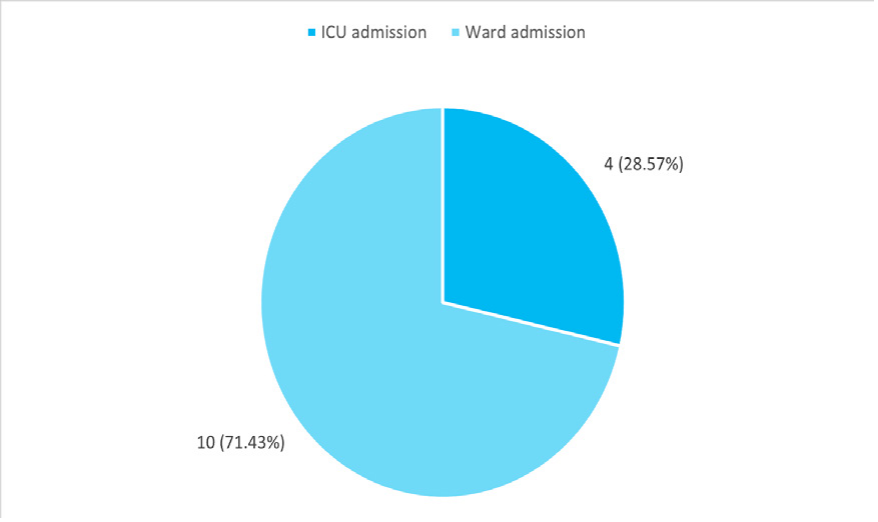
Mode of admission (n= 14).

Four (28.57%) patients were admitted to the Intensive Care Unit (ICU) whereas 10 (71.43%) patients were admitted to the general ward (Figure 1).

**Figure 2:**
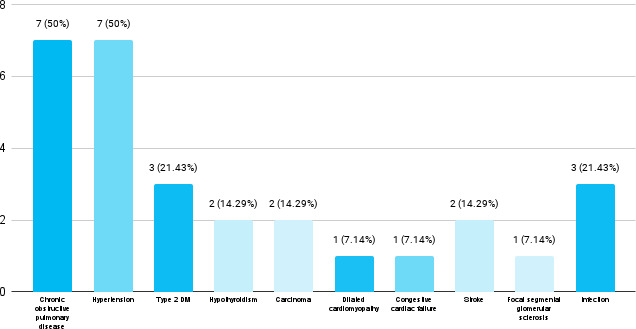
Comorbidities present in the community-acquired pneumonia patients (n= 14).

Among 14 patients, cardiovascular conditions were seen in 9 (64.28%) followed by chronic obstructive pulmonary disease which was seen in 7 (50%) as a comorbidity (Figure 2).

## DISCUSSION

The prevalence of community-acquired pneumonia among elderly patients was found to be 14 (3.64%) in our study, which is lower in comparison to previous similar studies. According to research conducted at a tertiary medical centre in the Philippines, the overall frequency of elderly patients hospitalized with community-acquired pneumonia was 5%.^9^

In our study, the mean age was found to be 70.57±10.21 years which is lower compared to other studies. Research conducted at multicenter hospitals in Spain discovered that the average age of patients was 76.3±7.3 years.^10^ Another research conducted at Fudan University in China found that the average age of elderly patients with community-acquired pneumonia was 79.8±8.8 years.^11^

The patients in this study consisted of 6 (42.86%) male patients and 8 (57.14%) female patients with a male-to-female ratio of 0.75 showing the preponderance of female patients. However, similar studies done in Spain and China showed the male to female ratio of 1.73 and 1.25 respectively.^10,11^

In our study, 9 (64.29%) had a history of smoking while 4 (28.57%) had a history of alcohol intake, while another study done in Spain revealed, 52% of community-acquired pneumonia had a history of smoking and 18% had a history of alcohol consumption.^10^ This study showed that 4 (28.57%) of patients required ICU admission whereas 10 (71.43%) of patients were admitted to the ward which is higher in comparison to other studies done. A study done in the United States showed that 22.4% of community-acquired pneumonia patients required ICU admission.^12^

In our study, the mean hospital stay among elderly community-acquired pneumonia patients was higher than in other studies and was found to be 7.42±3.41 days. A study done in the United States regarding the relative burden of community-acquired pneumonia hospitalisation in older adults showed an average hospital stay of 5.6 days.^13^

In our study, among elderly community-acquired pneumonia patients, cardiovascular conditions were seen in 9 (64.29%) of patients followed by chronic obstructive pulmonary disease as a comorbidity which is similar to other studies done. A similar study done in Spain showed that 44% had cardiovascular condition as a comorbidity followed by respiratory disease in 42% patients.^10^ Another research on community-acquired pneumonia in the elderly found that the most prevalent comorbidity was cardiovascular illness in 38% of the patients, followed by chronic obstructive pulmonary disease in 30%.^14^

In-hospital mortality among elderly community-acquired pneumonia in our study was 3 (21.42%). In a study done in China, in-hospital mortality was 19.8% among elderly community-acquired pneumonia patients.^11^

There are a few limitations in our study. This was done in a single hospital setting at a government tertiary referral care hospital in the nation's capital. As a result, the results might not be generalizable. Therefore, before making any use of the results, the study's limitations should be taken into account. The study excluded laboratory measurements and pneumonia severity indices that might have added information on the risk factors for death in older patients with community-acquired pneumonia.

## CONCLUSIONS

The prevalence of community-acquired pneumonia among the elderly in a tertiary care hospital was found to be lower compared to other studies conducted in similar settings.
